# Winter nitrification in ice-covered lakes

**DOI:** 10.1371/journal.pone.0224864

**Published:** 2019-11-07

**Authors:** Emily Cavaliere, Helen M. Baulch

**Affiliations:** University of Saskatchewan, School of Environment and Sustainability, Global Institute for Water Security, Saskatoon, Saskatchewan, Canada; University of Maine at Farmington, UNITED STATES

## Abstract

With changes in ice cover duration, nutrient loading, and anoxia risk, it is important to understand the mechanisms that control nitrogen cycling and oxygen depletion in lakes through winter. Current understanding is largely limited to description of changes in chemistry, with few measurements of the processes driving winter changes, how they differ across lakes, and how they are impacted by under-ice conditions. Nitrification is a process which consumes oxygen and ammonium (NH_4_^+^), and supplies nitrate (NO_3_^–^). To date, nitrification has been measured under ice cover in only two lakes globally. Here, we used ^15^NH_4_^+^ enrichment to measure rates of pelagic nitrification in thirteen water bodies in two ecozones. Our work demonstrates ecologically important rates of nitrification can occur despite low water temperatures, impacting NH_4_^+^, NO_3_^–^ and, most importantly, oxygen concentrations. However, high rates are not the norm. When, where and why is nitrification important in winter? We found that nitrification rates were highest in a eutrophic lake chain downstream of a wastewater treatment effluent (mean: 226.5 μg N L^-1^ d^-1^), and in a semi-saline prairie lake (110.0 μg N L^-1^ d^-1^). In the boreal shield, a eutrophic lake had nitrification rates exceeding those of an oligotrophic lake by 6-fold. Supplementing our results with literature data we found NH_4_^+^ concentrations were the strongest predictor of nitrification rates across lentic ecosystems in winter. Higher nitrification rates were associated with higher concentrations of NH_4_^+^, NO_3_^–^ and nitrous oxide (N_2_O). While more work is required to understand the switch between high and low nitrification rates and strengthen our understanding of winter nitrogen cycling, this work demonstrates that high nitrification rates can occur in winter.

## Introduction

**Fig 1:**
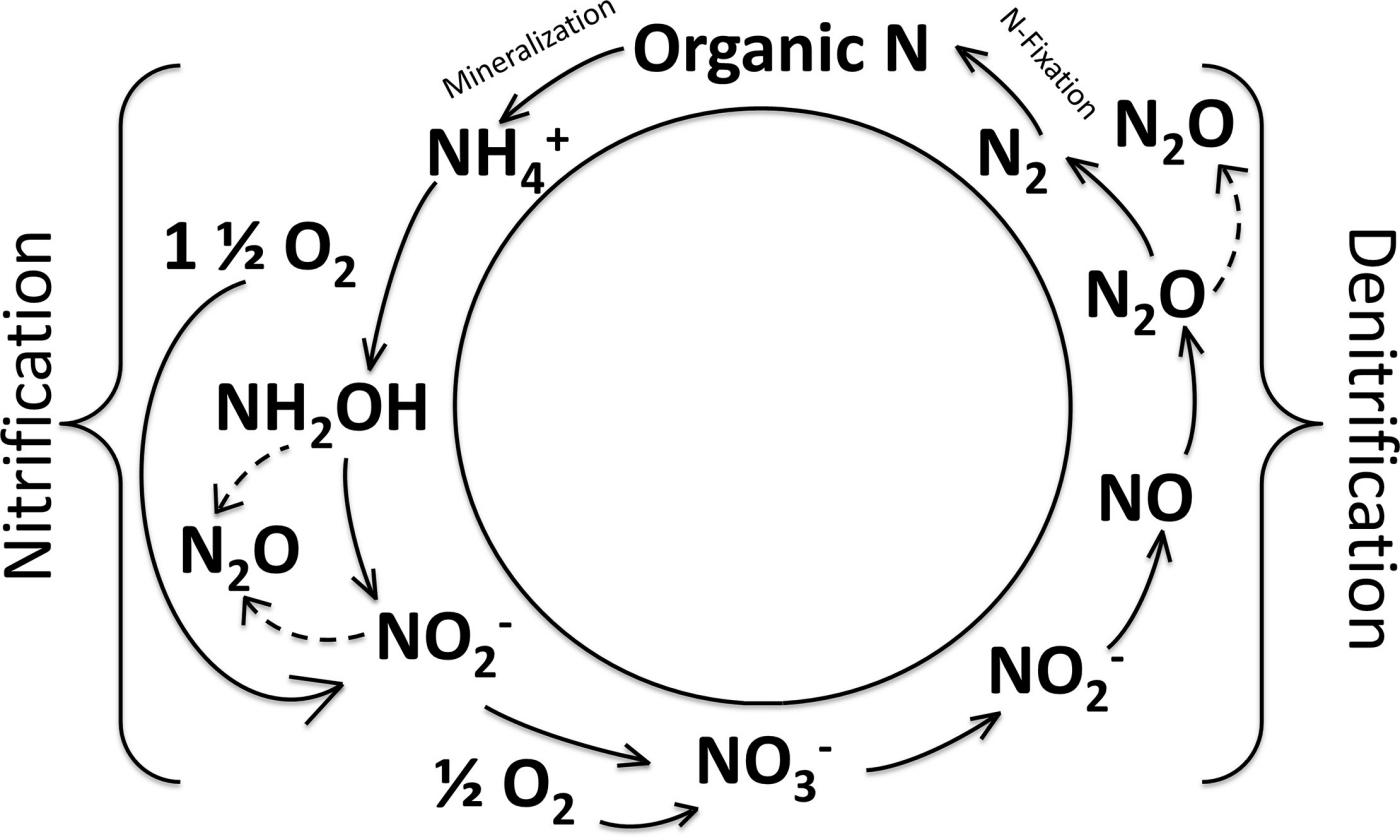
Schematic of the nitrogen cycle, featuring the microbial processes of nitrification (NH_4_^+^ to NO_2_^–^ and then to NO_3_^–^) and denitrification (NO_3_^–^ to NO_2_^–^, nitric oxide, N_2_O then to nitrogen gas). For every mole of NH_4_^+^ nitrified to NO_3_^–^, two moles of oxygen are consumed (Stoichiometric relationships collectively found in [5,6,10–13]). Note that the proportion of N_2_O released from nitrification and denitrification is highly variable as indicated by the dashed arrows [14]. Nitrogen assimilation, dissimilatory NO_3_^–^ reduction to NH_4_^+^ (DNRA) and anaerobic NH_4_^+^ oxidation (anammox) are excluded from the figure but may be important components of the nitrogen cycle [15].

Changes to the global nitrogen (N) cycle have led to significant increases in N inputs to rivers, lakes, oceans, and the atmosphere [1]. Strongly elevated nitrogen concentrations and associated ecological effects are shown in many aquatic ecosystems, often driven by runoff from intensive agriculture [2]. However, within freshwater ecosystems, some of the most acute impacts of nitrogen fertilization are seen at sewage outfalls–where high NH_4_^+^ concentrations are nitrified [3,4]. The process of nitrification is a microbially-mediated one, whereby NH_4_^+^ is oxidized to nitrite (NO_2_^–^) then to NO_3_^–^, (Fig 1; [5]). Nitrification leads to consumption of oxygen, which can be associated with fish kills [6,7]. In addition, because nitrification is the oxidation of NH_4_^+^, nitrification impacts the availability of different nitrogen species [8], which can affect phytoplankton taxa and productivity [9].

Nitrate generated from nitrification as a product, is a substrate for denitrification (Fig 1). As such, nitrification can fuel the process of denitrification–a process which is considered an ecosystem service because it permanently removes fixed nitrogen. However, both nitrification and denitrification contribute to the production and emission of nitrous oxide (N_2_O), a greenhouse gas and contributor to stratospheric ozone depletion (Fig 1; [16,17]). Recent work shows that N_2_O supersaturation is common under ice [18] and is indicative of active nitrogen cycling in winter. This conclusion of active N-cycling under ice is further supported by recent research demonstrating that winter denitrification rates are similar to those observed in warmer summer months [17], and evidence from Wisconsin lakes that nitrification contributes to winter NO_3_^–^ production and oxygen depletion [6,19].

Nitrification may be the most important process in the nitrogen cycle to understand in winter due to its role in winter oxygen decline [6,20,21]. Substantive increases in winter NO_3_^–^ in Wisconsin lakes are indicative of nitrification and may drive up to 25% of the observed oxygen decline [6,19]. Nitrification can be stimulated by increased substrate availability [22], suggesting that the potential for higher NH_4_^+^ availability (e.g., due to limited competition from autotrophs), could contribute to enhanced nitrification in winter [19]. Despite knowledge that the nitrogen cycle can be active under cold conditions, our understanding of biogeochemical cycling in the ice covered period is still in its infancy.

There are major physical, chemical and biological changes that might be expected to impact nitrogen cycling in ice-covered lakes [23]. Ice cover isolates lakes from the atmosphere, which leads to increased risk of hypoxia or anoxia in shallow, and snow-covered water bodies, driven both by aerobic respiration and nitrification [6,21,24]. Low light penetration can limit autotrophic activity and nutrient uptake [25]. Respiration (or mineralization) of organic matter continues, producing NH_4_^+^, which can either contribute to build up of this solute, or NH_4_^+^ may be consumed, for example, by nitrification [6,23,26]. The low light conditions in winter may also be advantageous to nitrifiers (where adequate oxygen is available) because light can inhibit nitrification [25,27]. However, the importance of this effect in winter is not known, as it is species specific, wave-length specific and dose dependent [28].

Winter conditions may also slow down or inhibit nitrification. Low oxygen availability in winter [29] constrains nitrification rates in some ecosystems [30]. Typically, low temperatures are associated with low rates of microbial activity, and this is true for nitrifiers. Increasing temperatures have a positive impact on rates of nitrification, particularly at moderate temperatures (10 to 35°C; [31,32]). However, there is some evidence from work in the Arctic that nitrifying microbes can adapt to cold temperatures [33]. Finally, methane (CH_4_) accumulation during winter [34,35] is also potentially important in controlling nitrification rates. Nitrifiers and methanotrophs have similar monooxygenases [36] and as a result, methanotrophs can oxidize NH_4_^+^, much like nitrifiers can oxidize CH_4_. CH_4_ availability may affect nitrification rates via competitive inhibition [36,37].

**Fig 2:**
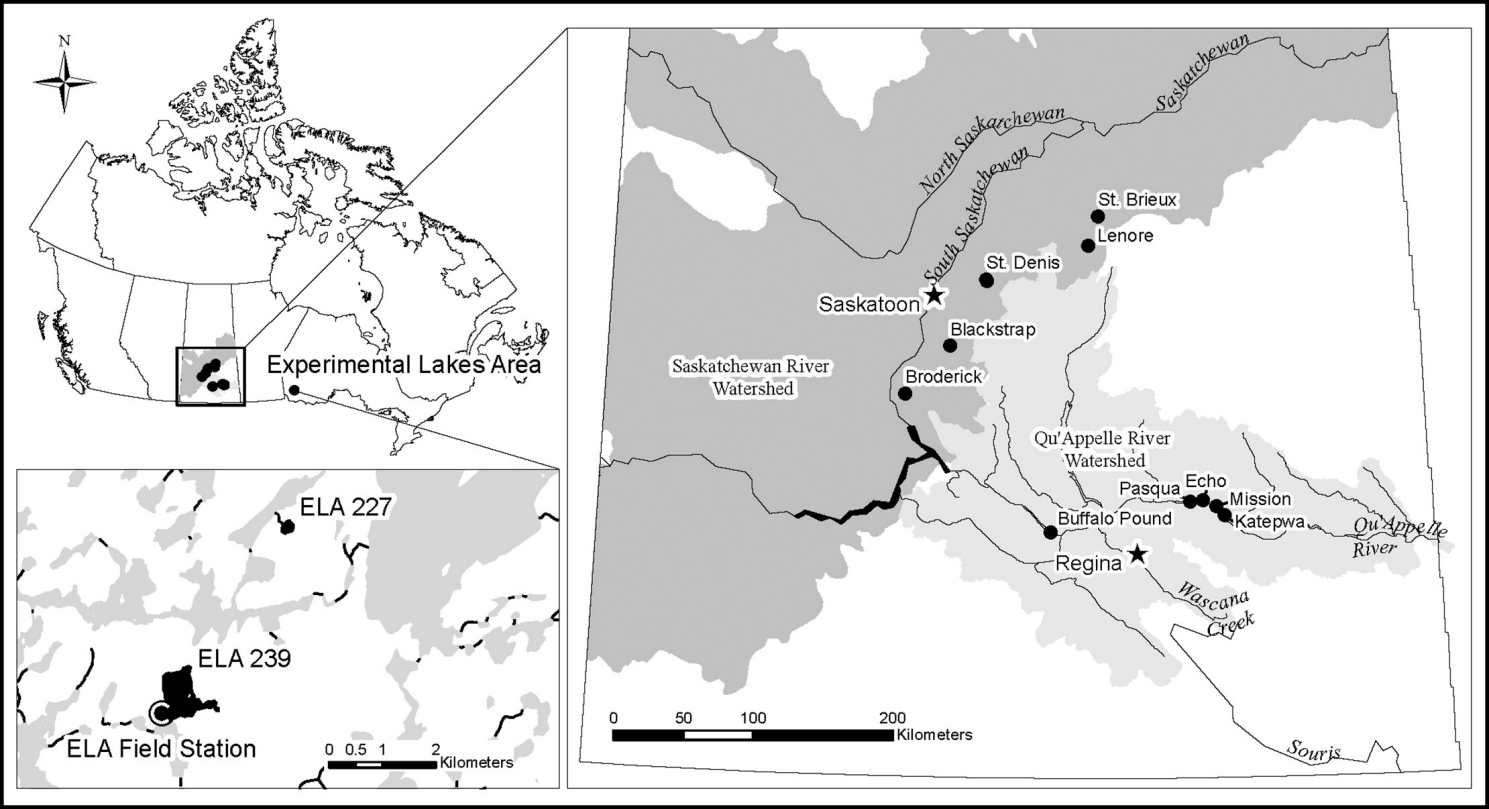
Map of Canada with overlays of Saskatchewan and Ontario study sites (map courtesy of Rosa Brannen). All Saskatchewan sites are in the prairie ecozone, while the Experimental Lakes area sites are in the boreal shield ecozone [42].

Quantifying the multitude of factors affecting nitrification is important to understanding current hypoxia risk, nitrogen cycling and future changes. Yet, few process-based measurements of nitrogen cycling in winter have been reported. Currently our understanding of nitrification in winter is limited to direct measurements in Lake St. George, Ontario, Table 1 [21], and in Lake Croche, Québec, Canada [38], an isotope-based study of nitrification in Smith Lake, Alaska [39], measurements in the cold, but ice-free Lake Superior (Table 2; [40]) and estimates of nitrification and NO_3_^–^ accumulation under ice from lakes in Wisconsin (Table 1 [6,19]). All of these studies suggest that nitrification can be important to oxygen decline in winter, yet this is a small number of measurements compared to the millions of seasonally ice-covered lakes globally [41]. Here we ask the questions: 1) What are pelagic nitrification rates under ice? 2) What factors are associated with high rates of winter nitrification? 3) Can winter nitrification be a significant mechanism for oxygen depletion under ice? and 4) Are nitrification rates correlated with N_2_O accumulation under ice? We measured nitrification rates from thirteen lakes, ponds, and reservoirs in Saskatchewan and northern Ontario, Canada (Fig 2) which cross two northern ecozones to answer these questions, and supplemented our measurements with data from the literature.

**Table 1:** Winter nitrification rates and associated data for this study (under ice cover) and for Lake St. George (near surface at 2 m depth, under ice cover; [21]); for surface nitrification rates in Lake Croche (ice-covered; [38]); and for surface estimates of nitrate accumulation in Wisconsin lakes part of the North Temperate Lakes Long-Term Ecological Research (NTL- LTER) study (ice-covered, 30 years of accumulated data; [19]). Values below limits of quantitation (LOQ) for nitrification rates are reported, including negative values (following [40]) and sample-specific LOQ (as described in Methods and calculated as per [43,44]) are reported.

Location	Nitrification Rate	Nitrification Rate LOQ	Ammonium	Nitrate	Oxygen	pH	Specific Conductance	N_2_O	CH_4_	Date
	(μg N L^-1^d^-1^)	(μg N L^-1^d^-1^)	(μg N L^-1^)	(μg N L^-1^)	(mg L^-1^)		(μS cm^-1^)	(% Saturation)	(% Saturation)	
Winter Ice Cover										
Blackstrap Reservoir	-1.7*	4.9 x 10^−3^	<86**	<57**	7.6	5.7	1237	134.8	97.6	05-Mar-15
Buffalo Pound Lake	-4.0*	4.0 x 10^−3^	<86**	<57**	12.5	7.6	1185	184.8	246.1	10-Mar-15
Echo Lake	1.2	7.0 x 10^−2^	366	171	8.3	8.6	1726	213.7	241.5	02-Feb-16
Katepwa Lake	32	2.7 x 10^−2^	<86**	620	9.9	6.8	1566	636.2	414.9	10-Mar-15
Lenore Lake	-1.7*	6.8 x 10^−3^	263	200	7.5	8.4	3758	253	259.3	25-Mar-15
Mission Lake	2.1	1.1 x 10^−1^	452	129	7.6	8.5	1790	195.4	282.1	02-Feb-16
Pasqua Lake	870.7	3.2 x 10^−3^	1650	532	8.6	7.3	2180	671	178	10-Mar-15
St. Denis Pond 1	-0.042*	3.2 x 10^−3^	<86	<57**	10	6.9	1536	132.9	1109.3	16-Apr-15
St. Denis Pond 5338	-0.044*	3.1 x 10^−3^	<86**	<57**	15	8.2	2963	135.7	1027.6	16-Apr-15
St. Denis Pond 90	-0.1*	3.6 x 10^−3^	<86**	<57**	14.5	8.6	1648	109.1	1650.2	16-Apr-15
St. Brieux Lake	110	1.8 x 10^−3^	528	310	2	7.8	3589	515.6	579.8	25-Mar-15
ELA Lake 227	0.14	4.6 x 10^−5^	516	314	5.84	6	26	NA	NA	14-Mar-16
ELA Lake 239	0.022	1.2 x 10^−4^	636	145	13.7	6.6	30	NA	NA	14-Mar-16
Lake St. George, Ontario	22.8	NA	467	540	9.3	NA	NA	NA	NA	27-Feb-80
Lake St. George, Ontario	12.2	NA	390	550	8.1	NA	NA	NA	NA	06-Mar-80
Lake St. George, Ontario	4.5	NA	65	1230	1.3	NA	NA	NA	NA	24-Feb-82
Lake St. George, Ontario	1.3	NA	39	1191	0.8	NA	NA	NA	NA	0Mar-82
Lake St. George, Ontario	10.5	NA	490	210	10.2	NA	NA	NA	NA	09-Feb-83
Lake Croche	4.7	NA	38	19	NA	NA	NA	NA	NA	01-Feb-12
Lake Croche	2.6	NA	38	27	NA	NA	NA	NA	NA	01-Mar-12
Allequash Lake, Wisconsin	0.58^ƒ^	NA	NA	NA	NA	NA	NA	NA	NA	30 years of data
Big Musky Lake, Wisconsin	0.55^ƒ^	NA	NA	NA	NA	NA	NA	NA	NA	30 years of data
Crystal Lake, Wisconsin	0.39^ƒ^	NA	NA	NA	NA	NA	NA	NA	NA	30 years of data
Sparkling Lake, Wisconsin	0.45^ƒ^	NA	NA	NA	NA	NA	NA	NA	NA	30 years of data
Trout Lake, Wisconsin	0.87^ƒ^	NA	NA	NA	NA	NA	NA	NA	NA	30 years of data

*Indicates less than associated Limits of Quantitation for that sample.

**Indicates that sample concentrations were below method detection limits of 86 and 57 μg N L^-1^ for NH_4_^+^ and NO_3_^–^, respectively. NA indicates data were not reported.

^ƒ^Indicates data from [6] are estimated from winter nitrate accumulation and likely underestimated.

**Table 2:** Winter nitrification rates and associated data for Lake Superior (near surface at 2 m depth, in winter but without ice-cover; [40]). Summer values of nitrification rates are reported for the two lakes (Western Basin of Lake Superior and Lake Croche) where cross-season study has been performed. Values below limits of quantitation (LOQ) for nitrification rates are reported, including negative values (following [40]) and sample-specific LOQ (as described in Methods and calculated as per [43,44]) are reported.

Location	Nitrification Rate	Nitrification Rate LOQ	Ammonium	Nitrate	Oxygen	pH	Specific Conductance	N_2_O	CH_4_	Date
	(μg N L^-1^d^-1^)	(μg N L^-1^d^-1^)	(μg N L^-1^)	(μg N L^-1^)	(mg L^-1^)		(μS cm^-1^)	(% Saturation)	(% Saturation)	
Winter No Ice										
Western Basin of Lake Superior	0.34	NA	2.55	NA	NA	NA	NA	NA	NA	11-Nov-09
Western Basin of Lake Superior	0.03	NA	2.25	NA	NA	NA	NA	NA	NA	20-Mar-11
Summer										
Western Basin of Lake Superior	0.11	NA	1.8							27-Jun-10
Western Basin of Lake Superior	0.33	NA	4.35	NA	NA	NA	NA	NA	NA	19-Aug-10
Lake Croche	0.81	NA	22.5	45.6	NA	NA	NA	NA	NA	01-May-12
Lake Croche	0.49	NA	7	28	NA	NA	NA	NA	NA	27-May-12
Minimum	0.022	4.6 x 10^−5^	2.3	19	2	5.7	26	109.1	97.6	
Median	1.3	3.6 x 10^−3^	86	186	8.45	7.6	1648	195.4	282.1	
Maximum	870.7	1.1 x 10^−1^	1650	1230	15	8.6	3758	671	1650.2	

*Indicates less than associated Limits of Quantitation for that sample.

**Indicates that sample concentrations were below method detection limits of 86 and 57 μg N L-1 for NH_4_^+^ and NO_3_^–^, respectively. NA indicates data were not reported.

## Methods

### Study sites and sampling

Our study sites included 11 Saskatchewan lakes, ponds and reservoirs in the prairie ecozone (Fig 2) and two lakes at the International Institute for Sustainable Development- Experimental Lakes Area (IISD-ELA) in northwestern Ontario, Canada (Boreal Shield; Fig 2). Samples were typically collected in mid-late winter, with the majority of samples collected in March or February (Table 1). The St. Denis ponds were sampled in April during the melt period. The Saskatchewan water bodies include sites that are sources of drinking water, provide wildlife habitat and are important sites for recreation [45–47]. Buffalo Pound is a reservoir that is part of the Qu’Appelle system, upstream of Regina. Further along the Qu’Appelle chain are Pasqua, Echo, Mission and Katepwa lakes. These four lakes are impacted by wastewater discharge from the upstream city of Regina as well as nearby agriculture [3]. Three other water bodies are ponds in the St. Denis National Wildlife Area in a series of periodically connected ponds. A permit for sampling of these ponds was obtained by author Dr. Helen Baulch for 2013 through 2016 from Environment Canada and the Canadian Wildlife Service (permit numbers: 2012–085, 2015–001). Blackstrap is a reservoir, and St. Brieux and Lenore are interconnected lakes. These Saskatchewan water bodies are naturally mesotrophic or eutrophic [45], and are impacted by human activities including agricultural land use and wastewater inputs. They face many challenges due to poor water quality, including oxygen depletion and degraded source water quality [3,48,49]. In contrast, Lake 239 at the ELA is a low phosphorus and low chlorophyll concentration (oligotrophic) lake while Lake 227 is naturally oligotrophic, but has been the subject of a multi-decade nutrient enrichment experiment altering the nutrient chemistry and trophic status over the past 40 years [50,51]. From 1969 to 1989 both N and phosphorus (P) were added to Lake 227 then from 1990 to 2005 only P was added [51].

Water samples and samples of dissolved gases were obtained in 2015 and 2016 by boring through the ice in each of the 11 Saskatchewan study sites. Samples for chemical analyses and nitrification experiments were obtained via peristaltic pump from a depth of 0.5 m below the ice-water interface into a plastic, acid-washed 20-L carboy in a heated tent (to prevent freezing in temperatures that frequently reached -30°C). *In situ* oxygen, temperature, pH, and specific conductance profiles at the time of sampling were collected using the YSI 556 Multi Probe System (YSI Environmental, Yellow Springs, OH) for all water bodies except St. Brieux and Lenore where the YSI ProPlus was used, courtesy of Dr. John-Mark Davies (Water Security Agency). Water and gas samples for the three St. Denis sites were collected via peristaltic pump, as well, but samples were obtained from shore due to unsafe ice conditions. Sampling of these sites was achieved by anchoring hoses in the ice ~ 5-10m off shore (over ~2m of water) and using the pump and hoses to transfer water to the shore, where water, gas, and YSI measurements could be taken. This approach, necessitated by safety concerns, may contribute to elevated oxygen measurements at these sites. Dissolved gases (CH_4_ and N_2_O) were sampled via peristaltic pump by using headspace equilibrations after overfilling with sample water a 1.2-L glass bottle [52] in the Saskatchewan Lakes. Lakes 227 and 239 at the Experimental Lakes Area were sampled in a similar fashion for water, but were not sampled for CH_4_ or N_2_O. Experimental Lakes Area samples were obtained in March of 2016 by IISD-ELA (Ken Sandilands, biologist).

### Laboratory and experimental methods

Water samples were protected from freezing and filtered upon return to the laboratory through pre-rinsed 0.2-μm polycarbonate filters (A.M.D. Manufacturing, Mississauga, Ontario) under low vacuum pressure. Subsamples of filtered water samples were analyzed for NO_3_^–^ and nitrite (NO_3_^–^ and NO_2_^–^ EPA method 353.2, hereafter referred to as NO_3_^–^), NH_4_^+^ (EPA 350.1), soluble reactive phosphorus (EPA 365.1), sulfate (Standard Method 426C) and alkalinity (EPA method 310.2) using the SmartChem 170 autoanalyzer (Westco Scientific Instruments, Inc., Brookfield, CT). Water samples were analyzed for NH_4_^+^ and soluble reactive phosphorus within 24 h or less while water samples for sulfate and NO_3_^–^ analyses were frozen after filtering, thawed and then analyzed. Alkalinity was measured on refrigerated filtered water. The water samples from ELA were analyzed upon receiving the shipped samples (within 3 days of sampling).

Headspace gas samples were analyzed for N_2_O and CH_4_, in duplicate, using the Scion 456 Gas Chromatograph (Bruker Ltd.). A micro-electron capture detector (ECD) was used to measure N_2_O and the flame ionization detector (FID) was used to measure CH_4_. Dissolved N_2_O and CH_4_ concentrations were calculated using standard solubility equations [53]. For semi-saline systems (St. Denis, St. Brieux and Lenore lakes), we calculated ionic salinity (as per [54]) due to the dominance of sulfate, calcium, magnesium and sodium ions. Filtered water samples were analyzed for ions using Inductively Coupled Plasma–Optical Emission Spectrometry, Department of Geology, University of Saskatchewan. These corrected salinity values were then used to determine dissolved concentrations and solubility of N_2_O [53] and CH_4_ [55].

### Nitrification experiment

Nitrification experiments were carried out as follows and as outlined in [56] adapted from [57]. In brief, water samples were analyzed for NH_4_^+^ on the SmartChem 170 Autoanalyzer, as noted previously (EPA 350.1). Unfiltered water samples were placed in Wheaton B.O.D. bottles, and samples were fortified with a spike of ^15^N-enriched NH_4_Cl (Sigma-Aldrich; 98 atom % ^15^N, Lot # MBBB2704V) at a concentration equivalent to 10% of the measured NH_4_^+^ concentration. If NH_4_^+^ concentrations were below the MDL, historical concentrations (last winter measurement; typically the previous month) were used as a reference for the addition of 10% of *in situ* for ^15^N- NH_4_^+^ addition. For later calculations, the NH_4_^+^ MDL was used to approximate *in situ* concentrations. Samples were typically incubated at 4°C for 60 h in the dark on a VWR DS-500 Orbital Shaker (Henry Troemner LLC, New Jersey). The 60 h incubation time was designed to account for decreased activity at lower temperatures and to help maximize sensitivity of the method. However, the longer incubation time may allow isotope recycling, where the supplied ^15^NH_4_^+^ supplied is re-released, resulting in potential underestimation of nitrification rates. We performed a subset of analyses using 24h incubations in addition to our standard 60h incubations (results are reported in S1 Table) to assess whether incubation time or recycling. Although we found no statistically significant difference between the two periods of incubation (Wilcox-Mann-Whitney Test, p-value = 0.125, 6 DF; S1 Table), the data suggest a potential impact of incubation time (S1 Table). As a result, 60 h rates presented here should be viewed as a minimum nitrification rates. After incubation, the enriched water samples were filtered under low vacuum pressure through 0.2-μm polycarbonate filters to halt bacterial activity. The ^15^N-enriched water samples were used to calculate nitrification rates after ^15^NO_3_^–^ was recovered.

### Nitrate recovery

Nitrification generates ^15^NO_3_^–^ (and ^14^NO_3_^–^) in the experiments. In order to recover ^15^NO_3_^–^ from the sample water, the water samples were processed as follows using the NH_4_^+^ diffusion disk method outlined in [58], with minor alterations. The post-incubation filtrate was poured into sealable media bottles and magnesium oxide was added (MgO; combusted at 650°C for 4 h). Samples were incubated at 65°C for five days to help decompose dissolved organic nitrogen (DON) to NH_4_^+^_._ The MgO enhances conversion of NH_4_^+^ to ammonia gas (NH_3_) in the high pH conditions (pH ~9.7; [58]). This step removes labeled ^15^NH_4_^+^/^15^NH_3_ that was not converted to NO_3_^-^ from the media bottles to prevent trapping later by the NH_4_^+^ diffusion disk [56,58]. Several studies use one or more of these techniques to remove either natural abundance or enriched ^15^N as ^15^NH_4_^+^ or ^15^N-DON–including in several systems with high natural nutrients [59], or human impacted systems, including freshwater streams [60], estuaries [61–63] and a saline lake [56].

During the five day processing period, the media bottles were vented daily to release nitrogen gases. Next, the water samples were boiled in the media bottles to further remove NH_4_^+^ and reduce volume to below 100 mL. A NO_3_^–^ spike of unlabeled NO_3_^–^ was added if previously measured *in situ* NO_3_^–^ concentrations were too low for analysis. The water samples were then adjusted to volume (100 mL) with deionized, distilled water. The water samples were then transferred back to the media bottles and sodium chloride was added (5 g) under recommendation by Dr. Sigman (pers. comm. D. M. Sigman; March 5, 2015). This modification was required as the original protocol was developed for seawater [58], hence sodium chloride was added to avoid osmotic pressure on the diffusion packets and subsequent disintegration.

To recover the enriched ^15^NO_3_^–^ (from nitrification) onto the diffusion disk, it must first be converted to ^15^NH_4_^+^. The diffusion disk (Teflon NH_4_^+^ diffusion disk packet, constructed using glass fiber filter paper sealed in Teflon tape with KHSO_4_) was added to the media bottles with Devarda’s alloy (100 mg Devarda’s Metal Alloy; Fisher Scientific; Lot 137926) and bottles were sealed. This alloy reduces NO_3_^–^ to NH_4_^+^, captured in the diffusion packet where the NH_4_^+^ ions are converted to ammonia ions by the KHSO_4_ and where it is kept in that form until analysis. The samples were incubated at 65°C for four days. Samples were then shaken on a VWR DS-500 Orbital Shaker (Henry Troemner LLC, New Jersey) for 7 days at 60 rpm. The Teflon packets were removed, rinsed in 10% HCl and then rinsed in deionized, distilled water and stored in scintillation vials until they were prepared for shipment by placing the diffusion disk in tin capsules. Samples were analyzed for ^15^N using an Elementar Vario EL Cube or Micro Cube elemental analyzer (Elementar Analysensysteme GmbH, Hanau, Germany) which was interfaced to a PDZ Europa 20–20 isotope ratio mass spectrometer (Sercon Ltd., Cheshire, UK) at the University of California Davis Stable Isotope Facility (http://stableisotopefacility.ucdavis.edu/).

Although DON can impact recovery of ^15^N [59–61,63], our tests showed no bias affected with organic matter addition. Specifically, we tested whether the addition of organic matter (approximately 3.2 mg C L^-1^) impacted diffusion disk efficacy (recovery of ^15^NH_4_^+^) in water obtained from Blackstrap Lake. Recovery was not affected by organic nitrogen addition under either elevated organic matter (addition of approximately 3.2 mg C L^-1^; C:N of 116:1) or natural organic matter concentrations. In an experiment of three treatments of NO_3_^-^, NH_4_^+^, and NH_4_^+^ and carbon additions, the treatments resulted in similar recovery on diffusion disk via analysis on an Elemental Analyzer (analysis of variance, 4 DF, p = 0.373). Finally, we compared potential nitrification rates in Blackstrap using both the diffusion disk method (modified to measure N mass on Elemental Analyzer, Vario Micro Cube, University of Saskatchewan [58]) and the denitrification recovery of NO_3_^-^ method at the University of California Davis Stable Isotope Facility (http://stableisotopefacility.ucdavis.edu/; [64]), and results were indicative of similar results. Average rates of 534.6 and 532.4 μg N L^-1^ d^-1^ for diffusion disk and denitrification methods respectively, when treated with 1 mg NH_4_^+^-N L^-1^mg/L (10% of which was labeled with ^15^N).

### Data and statistical analysis

Nitrification rates were calculated as per Sigman et al. 1997 [58]. After corrections were made for natural abundance and unlabeled N (as NO_3_^–^ spike) and N addition due to Devarda’s Alloy (as per [58]), 46% of rates were below measurement thresholds. We calculated the limits of quantitation (LOQ) as follows: Using standard deviations of the ^15^N (atom %) of the enriched samples (specific for each analysis), a method detection limit (MDL) was calculated by multiplying the standard deviation by Student t Distribution quantile (specific degrees of freedom). Then for each specific nitrification rate calculation we calculated the associated ^15^N in micrograms (MDL in ^15^N (atom %) × total N mass) for each sample and used that mass to calculate a minimum detectable rate based off the volume and incubation time for that sample. These LOQ rates range from 4.6 x 10^−5^ to 0.11 μg N L^-1^ d^-1^, and all further statistical analyses used the highest LOQ rate (0.11 μg N L^-1^ d^-1^) in place of nitrification rates when they were lower than 0.11 μg N L^-1^ d^-1^. We assume that the low rates (< sample specific LOQ, in Table 1, reported with *) are due to low, un-measurable nitrification rates rather than because of nitrogen recycling and possible underestimation of rates due to this recycling.

Due to the non-normal nature of the data, non-parametric tests were performed in R version 3.4.1 [65]. In order to assess links among different measured variables, a principal component analysis (PCA) was used [65]. The PCA shows how strongly related variables are by the proximity of the vectors–the more closely two (or more) variables are related–the closer those vectors will be in matching vector length and angle. To assess the measured variables (pH, oxygen, NO_3_^–^, and NH_4_^+^ concentrations, percent saturation of N_2_O and CH_4_) that could predict nitrification rates, linear model permutations (lmp) were used [66]. To determine the best fit model, a general linear model was used to determine which set of variables make up the best model for predicting nitrification rates based on lowest (or best fit) AIC (Akaike’s Information Criterion–AIC) for each model permutation. Next we supplemented our data with all available winter nitrification rate data, which included results from Lake Superior (a non-ice covered lake; [40]), Lake St. George (ice-covered; [21]) and Lake Croche (ice-covered; [38]) to assess relationships between NH_4_^+^ concentrations and nitrification rates. Finally, we divided the data into low (< 1.1 x 10^−1^ μg N L^-1^ d^-1^) and higher (> 1.1 x 10^−1^ μg N L^-1^ d^-1^) nitrification rates and used Signed Rank Mann-Whitney tests to understand the differences between these groups of data (wilcox.test; [65]). This threshold value was selected because it represents the highest sample-specific LOQ (Table 1), and provides a reasonable separation between rates deemed to have little or no impact on nitrogen chemistry and oxygen consumption, and rates with a potentially important influence. To determine if inclusion of 24-hr incubation nitrification rates changed results, all analyses were done with and without them. The results for the linear model, N species comparison and PCA are the same, with only slight changes in R^2^ and P-values but not changes in significance or groupings (PCA) (S1 Table).

## Results

**Fig 3:**
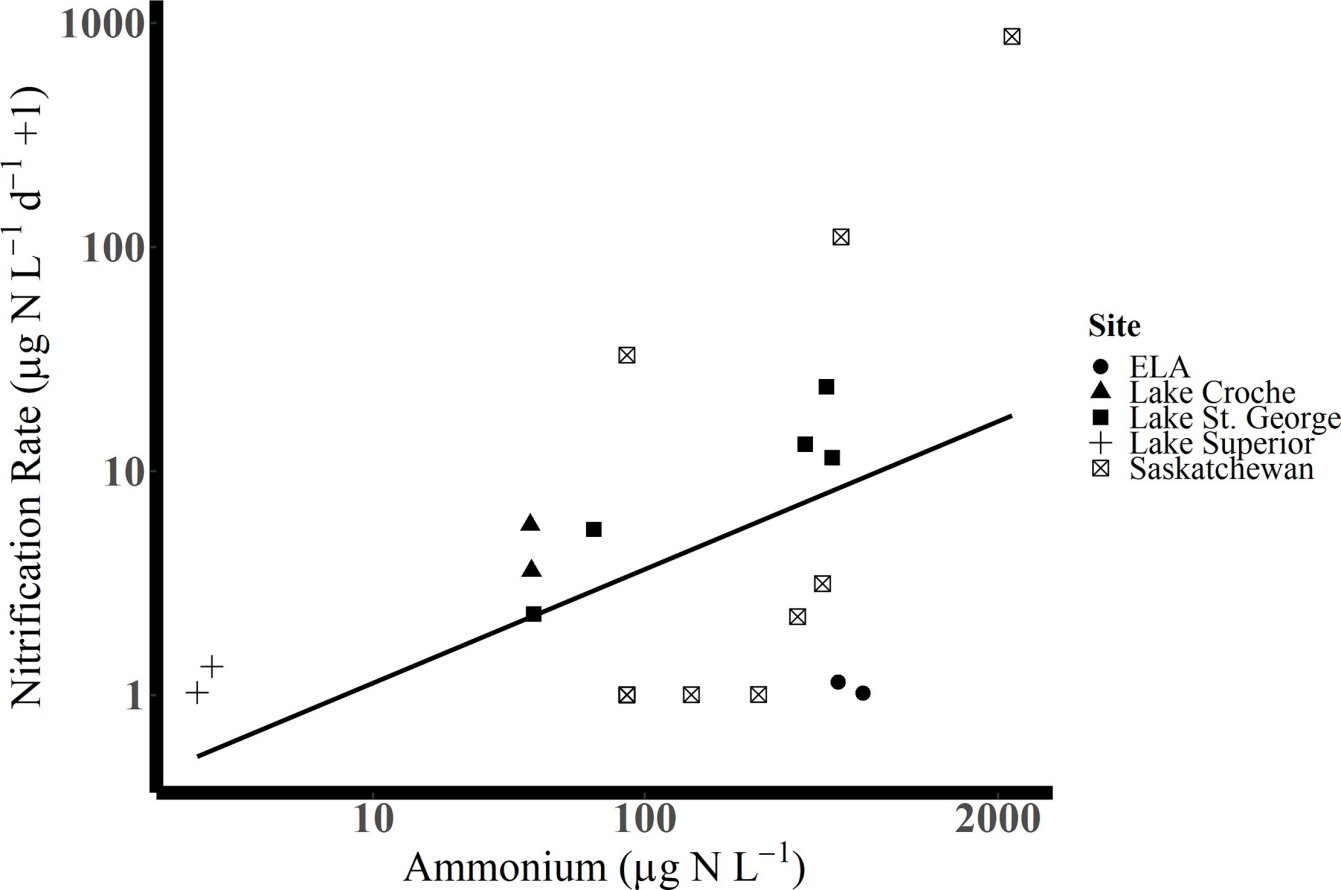
Relationship between nitrification rates and NH_4_^+^ concentrations for water bodies from this study (ELA and Saskatchewan) and other cold water measurements from Lake Superior (no ice-cover; [40]), Lake St. George (ice-covered; [21]) and Lake Croche (ice-covered; [38]). Note logged y-axis. The line plotted is the linear model (permutations) for all data from Tables 1 and 2. The linear model and statistics are presented in Table 3. Nitrification rates less than their sample specific LOQ are replaced by their LOQ.

**Fig 4:**
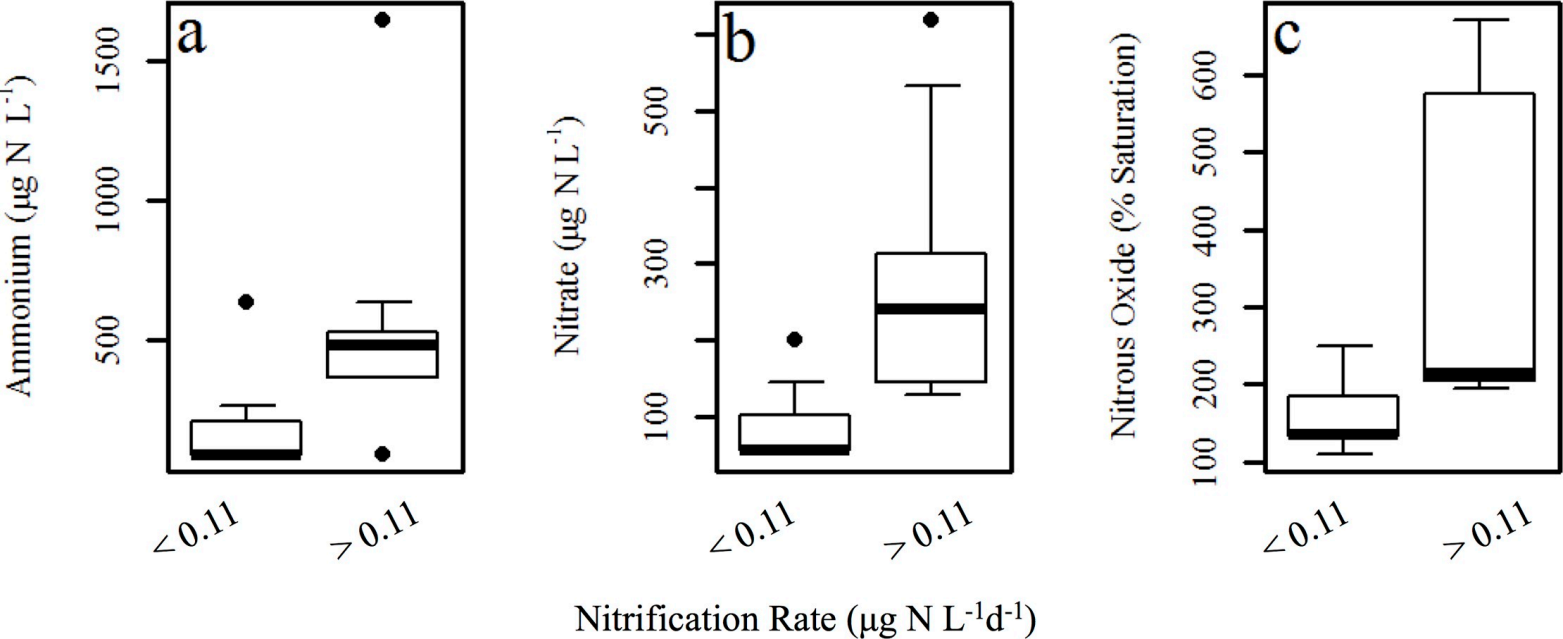
Concentrations of NH_4_^+^ and NO_3_^–^, and N_2_O percent saturation partitioned according to nitrification rates that are above or below 0.11 μg N L^-1^ d^-1^. There are significant differences between the two rate groups for all analyses (NH_**4**_^**+**^, NO_**3**_^**–**^, and N_**2**_O; Wilcox-Mann-Whitney test, P <0.05). The boxplot and whiskers encompass 95% of the data observed, data points outside of the box and whiskers are outliers. The box itself represents the first and third quartiles, and the center line is the median [65].

**Fig 5:**
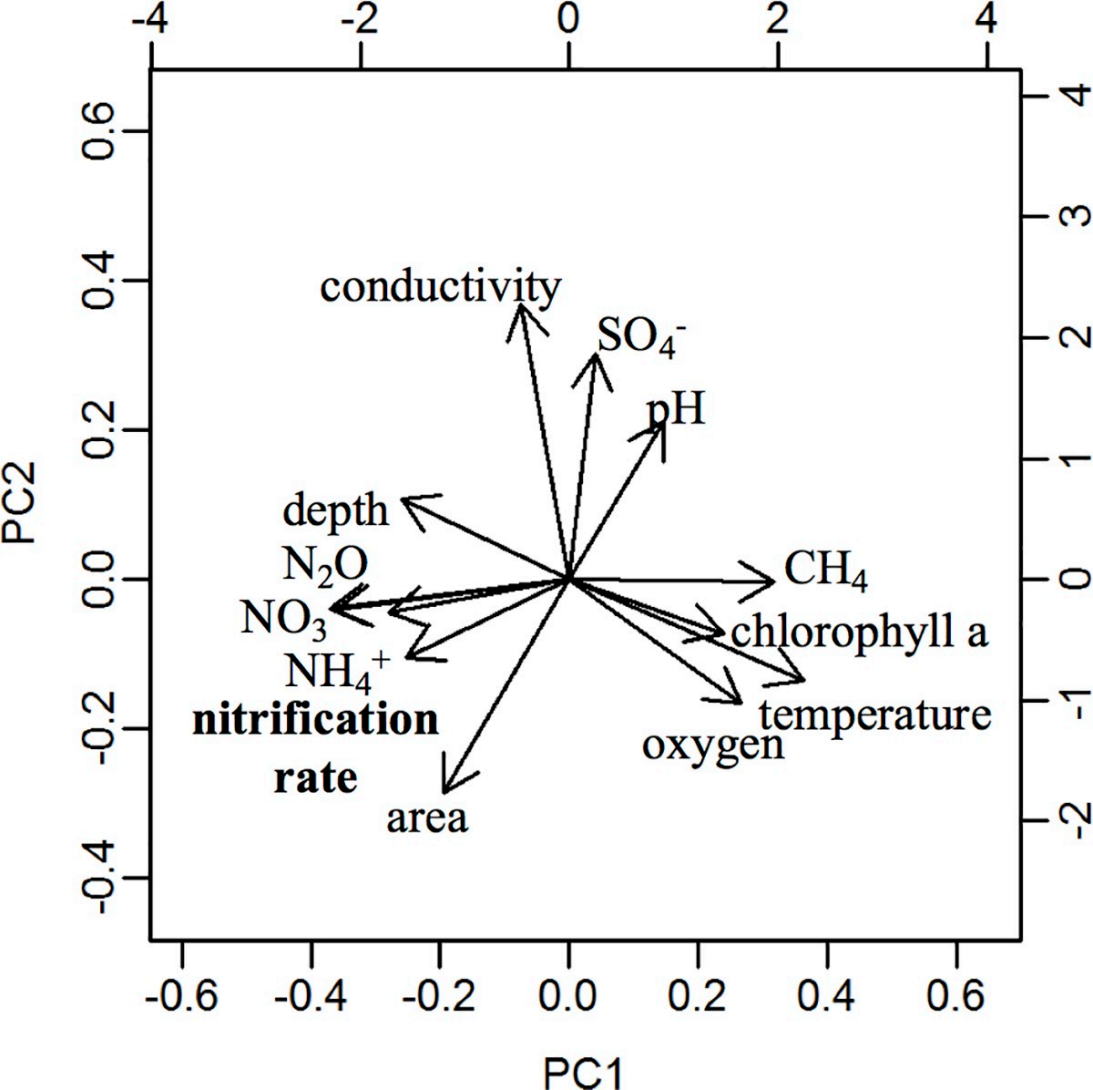
Principal component analysis showing the relationship between measured variables and nitrification rates. PC1 and PC2 account for 61% of the variance exhibited by the relationship among these variables. Within a PCA, the closer the component vectors are (angle and length) the more closely they are related. Note the association of nitrification rates with NO_**3**_^**–**^, N_**2**_O and NH_**4**_^**+**^, and of CH_**4**_ with chlorophyll and temperature.

Our highest measured winter nitrification rates exceeded past winter measurements in lentic freshwaters (Fig 3, Table 3), reaching rates as high as 870.7 μg N L^-1^d^-1^. Low, or unmeasurable rates were also very common–representing approximately half of our measurements (Fig 4). Partitioning the nitrification rates into low (< 1.1 × 10^−1^ μg N L^-1^ d^-1^) and higher (> 1.1 × 10^−1^ μg N L^-1^ d^-1^) rates revealed that nitrogen species differed across these groups. Higher nitrification rates were associated with elevated concentrations of NH_4_^+^, NO_3_^–^ and N_2_O saturation (Fig 4). Specifically, when nitrification rates were higher, (greater than 1.1 × 10^−1^ μg N L^-1^ d^-1^), median NH_4_^+^ concentrations were higher at 516 μg N L^-1^, while when rates were lower (less than 1.1 × 10^−1^ μg N L^-1^ d^-1^) median NH_4_^+^ concentrations were also markedly lower (86.0 μg N L^-1^; wilcox.test: P = 0.015, 11 DF, Fig 4A). The higher nitrification rates (>1.1 × 10^−1^ μg N L^-1^ d^-1^) were significantly associated with higher median NO_3_^–^ concentrations (310 μg N L^-1^) while lower (< 1.1 × 10^−1^ μg N L^-1^ d^-1^) nitrification rates were associated with lower median NO_3_^–^ (57 μg N L^-1^, wilcox.test: P = 0.011, 11 DF; Fig 4B). Finally, when nitrification rates were greater than the LOQ of 1.1 x 10^−1^ μg N L^-1^ d^-1^, median N_2_O saturation (516% saturation) was nearly four-fold greater than when rates were lower than the LOQ of 1.1 x 10^−1^ μg N L^-1^ d^-1^ (median 135% saturation; wilcox.test: P = 0.02, 9 DF; Fig 4C). Nitrous oxide was supersaturated in the surface waters of all lakes, ponds and reservoirs, ranging from 109 and 671% saturation. This same threshold (nitrification rates less than or greater than 1.1 × 10^−1^ μg N L^-1^ d^-1^) was not associated with differences in oxygen concentrations, CH_4_ percent saturation or pH (wilcox.test: P > 0.05). Across all data, the principal component analysis shows strong relationships among nitrification rates, concentrations of NH_4_^+^ and NO_3_^–^ and N_2_O percent saturation (capturing 61% of the variability of the data; Fig 5).

**Table 3:** Linear model relationships between nitrification rates (μg N L^-1^ d^-1^) and NH_4_^+^ concentrations (μg N L^-1^), using the linear permutations modeling approach. Literature data sources are noted in the caption of Fig 3.

Data usage	Linear model permutation	Model fit, significance, and degrees of freedom
All data (literature and this study)	Log(Nitrification rate+1) = 0.0012 × NH_4_^+^-N +0.62	Adjusted R^2^ = 0.47, P < 0.001, 20 DF
All data (literature and this study, excluding Pasqua Lake)	Log(Nitrification rate) = 0.0009 × NH_4_^+^-N– 0.51	Adjusted R^2^ = 0.06, P = 0.14, 19 DF
Only literature data	Log(Nitrification rate) = 0.0019 × NH_4_^+^-N + 0.68	Adjusted R^2^ = 0.70, P = 0.003, 7 DF

Data associated with published nitrification rates were somewhat more restricted, hence our combined analysis is restricted to assessing relationships between winter nitrification rates and NH_4_^+^ concentrations. This analysis showed that across all lakes (this study, and Lakes St. George, Superior and Croche) where pelagic nitrification has been measured in winter, rates were significantly predicted by NH_4_^+^ concentrations (lmp, P < 0.001; adjusted R^2^ = 0.47; 20 DF; Fig 3 and Table 3). Our data show a strong influence of the high nitrification rate observed at Pasqua Lake (without Pasqua: lmp, P = 0.14; adjusted R^2^ = 0.06; 19 DF; Table 3); however, we note that the literature data alone are indicative of a linear relationship (lmp; P = 0.003; adjusted R^2^ = 0.70; 7 DF; Table 3).

Nitrate concentrations and N_2_O % saturation were not linearly related to nitrification rates (lmp, P > 0.05), which, when combined with evidence of possible threshold effects (e.g., low nitrification rates associated with lower NO_3_^–^ concentrations and N_2_O % saturation), suggests non-linearity in the relationships, as might be expected where multiple factors (e.g., differential rates of nitrification and denitrification and yields of N_2_O) influence these parameters. Interestingly, CH_4_ percent saturation, winter temperature and oxygen concentrations do not appear to be related to nitrification rates (Fig 5, Table 3), although the range in variation in temperature was low, and oxygen concentrations were almost uniformly high.

Measurable nitrification rates within the prairies were predominantly found in Qu’Appelle lakes downstream of the outfall of the Regina Wastewater Treatment Plant. The outfall is released into Wascana Creek, then into the Qu’Appelle River, where the water enters, sequentially, Pasqua, Echo, Mission and Katepwa Lakes, lakes which are also significantly influenced by agricultural practices in their catchments. While these Qu’Appelle lakes showed significant nitrification rates, they were not directly related to lake position or distance from the wastewater treatment plant outfall. The saline St. Brieux Lake also showed measurable nitrification but the equally saline Lenore, which is presently connected to St. Brieux, did not. Within the boreal shield, nitrification rates were 6-fold greater in the experimentally eutrophied Lake 227 than the naturally oligotrophic Lake 239 despite both lakes having similarly high NH_4_^+^ concentrations.

## Discussion

Despite low temperatures, nitrification rates can be substantial in winter (Fig 3, Tables 1 and 2; [21]). Nitrification rates were also highly variable, suggesting some lakes may experience high rates of nitrification-related oxygen consumption at least in the short term, while nitrification will have little or no impact in others (Figs 1 and 3; [6,21]). In Pasqua Lake (influenced by upstream release of treated wastewater), NH_4_^+^ concentrations exceeded 1500 μg L^-1^, yet oxygen was available, and nitrification rates were exceptionally high (820 μg N L^-1^ d^-1^). These nitrification rates are greater than previously reported in winter, and this is despite evidence that 60 h measurements may underestimate true rates due to recycling (S1 Table). Ultimately, we provide further evidence suggesting that nitrification can be important under ice [6], and that low water temperatures do not preclude active nitrification in lakes [21,40] or other environments (e.g., biofilm reactor ponds; [67]).

Our results, combined with results from the literature, suggest that elevated NH_4_^+^ concentrations are typically associated with higher nitrification rates in winter (Table 3, Fig 3). This highlights the importance of management efforts to limit the export of NH_4_^+^ and organic matter in wastewater effluents, even during winter, a period which can pose significant technical challenges to wastewater treatment plants due to low temperature effects on treatment processes [68]. These technical challenges mean that higher concentrations of NH_4_^+^ are sometimes permitted in wastewater effluent during winter months [69]; however, it is worth noting that a recent plant upgrade at the Regina Wastewater Treatment Plant has been designed to reduce nitrogen loading, and similar changes in treatment have been implemented elsewhere. The need to limit the release of oxygen-demanding substrates has been known for decades [70,71]. However, there are subtleties here that necessitate consideration in the current debate regarding the need for nitrogen management of inland freshwaters [72,73]. Systems with higher nitrogen loads, even where effluents are nitrified and effluent oxygen demand is effectively controlled, may still have high mineralization rates leading to elevated NH_4_^+^ availability.

High rates of respiration or nitrification can put water bodies at risk for anoxia [21,74]. Within Saskatchewan water bodies, winter anoxia is relatively common, in part because of the shallow nature of many prairie lakes, long winters, and high benthic oxygen demand [75]. We can estimate the rate of oxygen depletion in surface waters due to nitrification using a mass ratio of 4.57:1 for O_2_ consumed per NO_3_^–^ produced (as per [6]). Scaling up our point measurements of pelagic nitrification leads to an estimate of a median nitrification oxygen demand (across study lakes) of 110 μg O_2_ L^-1^ per month (30 day month;548 μg O_2_ L^-1^ consumed over a ~5-month ice-cover period) which is substantial, but unlikely to markedly impact anoxia risk. We note that dark incubations may have favored enhanced rates of nitrification by removing any potential light inhibition [27,28,44]. Other studies of winter nitrification have reported variable impacts on oxygen depletion, from moderate influences (calculated from nitrification rates reported in Tables 1 and 2, range: 180–3120 μg O_2_ L^-1^ per month; [21]) to relatively small impact (range: 54–645 μg O_2_ L^-1^ per month; [6,38]).

While our measured median nitrification rates would have only a relatively small impact on oxygen, much higher rates of oxygen consumption may be observed, associated with higher nitrification rates (e.g., Pasqua Lake). Within Pasqua Lake, we may have captured a hot moment of nitrification. At the time of sampling in Pasqua Lake, oxygen concentrations were high, and oxygen inputs may also have been high, associated with low snow cover and potentially high primary productivity (e.g., [76]). Ultimately, we anticipate that the median seasonal rate of nitrification in Pasqua Lake is much lower than our point measurements suggest, and emphasize that high rates such as we observed in Pasqua cannot be sustained without substantial oxygen inputs to the lake via mixing. In contrast, St. Brieux had low oxygen concentrations, which could limit nitrification. Oxygen concentrations were less than 0.1 mg L^-1^; [77,78]) in the field and nitrification rates can become oxygen-limited between 0.1 and 1 mg L^-1^ O_2_ [77–79]; however, there is the potential that introduction of oxygen during sampling may have contributed to elevated rates observed in St. Brieux (nitrification rate of 110 μg N L^-1^d^-1^, see Table 1).

Winter in lakes is physically and biotically dynamic [23,25]. While key unknowns remain about biogeochemical cycling under ice, some of the most challenging questions may be about spatial and temporal variability and the ecological importance of short-term or small-scale pulses in microbial activity at an ecosystem scale [80]. Hot spots and hot moments are challenging to constrain in any season, but winter will bring unique challenges. Thus far, the limited work comparing pelagic nitrification rates in space, and within and across seasons suggests high variability in nitrification rates can occur (see Tables 1 and 2). For example, the depth maxima of nitrification rates in lakes may differ markedly over time [40]. In shallow waters, rates can be highly variable within winter of different years (e.g., 17-fold variation within Lake St. George; Table 1). High variability in nitrification rates across seasons (i.e., winter vs. summer) is also shown in some cases at a single depth (e.g., nearly 6-fold higher nitrification in winter compared to the open water season in Lake Croche [38]), although low winter-summer variation is also shown (Lake Superior [40]). Benthic nitrification may also be an important process if oxygen is present near the sediment-water interface [30], and rates of benthic nitrification may be similarly variable where transient mixing events occur [25,76], affecting oxygen delivery to the benthos. Ultimately, integrating approaches including: discrete measurements such as ours, with temporal changes in chemistry and natural abundance stable isotopes (e.g., [39]), and with sensor-based monitoring of oxygen and mixing, will help better integrate point measurements of rates through time and understand controls, and ecosystem-level implications.

Winter nitrification has the potential to impact the speciation of dissolved inorganic nitrogen at ice out, with possible impacts on phytoplankton communities. For example, diatom dominance may be favored when there is more available NO_3_^–^ and community dominance transitions to other species when NH_4_^+^: NO_3_^–^ increases [9,81,82]. Further, winter availability of NO_3_^–^ via nitrification also links nitrogen removal via denitrification, which remains active in winter (Fig 1; [17,83]). More work is required on the nitrogen cycle in its entirety to better understand factors controlling dissolved inorganic nitrogen concentrations and winter changes which may affect the spring bloom across the millions of temperate lakes globally [41]. We note that work employing shorter incubation times (minimizing isotope recycling), and alternative methods (i.e., ^15^N-NO_3_ isotope dilution method; [63,64]) may provide higher sensitivity for lower nitrification rates, improving our understanding of N cycling across many lakes in winter.

Winter appears to be an important time for N_2_O generation and build up under ice-cover [18]. N_2_O was supersaturated in all water bodies, which reflects active nitrogen cycling and the trapping of N_2_O under ice-cover, although we have periodically observed undersaturation near the sediments [17]. The observation that higher rates of nitrification are linked to higher N_2_O concentrations is not surprising, given N_2_O is produced as a result of nitrification (as well as via denitrification, Fig 1). Indeed, as much as 25% of NH_4_^+^ may be converted to N_2_O by nitrifying bacteria and ammonia oxidizing archaea [56,84], although yields are often much lower.

Low oxygen conditions are often associated with higher N_2_O concentrations, as observed here (Table 1; [84]). Low oxygen conditions affect nitrification rates, and are also critical to denitrification, another process which remains active under ice [17]. Importantly, the N_2_O yield of denitrification and nitrification may change through winter, as they are sensitive to a wide number of environmental factors including oxygen, temperature and pH, which can vary markedly through the ice cover period [85]. Because our measurements are more typical of mid-late winter conditions and not the ice-out period, more work is required to understand changes through winter affecting the speciation of dissolved nitrogen, and, processes affecting N_2_O production, and consumption prior to ice out. Given many lakes in later winter may have high NH_4_^+^ concentrations [75], this may increase the risk of a significant ice-out N_2_O emissions pulse [18].

Our work is also relevant to eutrophication management. While winter hypoxia risk is only one consideration in eutrophication management, this is an area where the effects of nitrogen management are not well understood, but may be particularly important. Within the prairie ecozone, nitrification rates were measurable only in two types of lakes: a hypoxic, semi-saline lake (St Brieux), and lakes downstream of a wastewater treatment plant (sequentially: Pasqua, Echo, Mission and Katepwa Lakes), that are situated in a catchment with extensive agricultural activity. Since nitrification rates from this study were measured in the Qu’Appelle chain of lakes in winters of 2015 and 2016, there has been a significant modification to the Wastewater Treatment Plant in Regina. The operation of nitrogen removal processes is expected to lead to reduced concentrations of nitrogen entering these water bodies. However, expectations for reduced nitrification rates should be tempered by evidence suggesting that eutrophication in the absence of nitrogen inputs may also impact nitrification rates. Within the boreal shield, nitrification rates of an experimentally eutrophied lake exceeded those of a naturally oligotrophic lake by 6-fold, although direct nitrogen additions to this lake ceased in 1990. The driver behind these differences is not known; however, we suggest assessment of the impact of eutrophication on organic matter quality and subsequent ammonification merits study, given nitrification is limited by substrate availability, as demonstrated here. Clearly more work is required to better understand the importance of both eutrophication and nitrogen management to nitrification rates in ice-covered ecosystems.

## Conclusion

Nitrification represents a key understudied control on lentic N_2_O budgets, and a control on the availability of different nitrogen species at ice out. Across lakes where nitrification has been measured in winter, nitrification rates are related to ammonium concentrations (Tables 1 and 2, Fig 3). This raises important questions about the importance of managing effluent ammonium in winter months–a period where cold temperatures can present technical challenges to treatment plants, but ecological sensitivity can be high due to elevated anoxia risk in ice covered ecosystems. We report an extremely high rate of nitrification in an ecosystem where high ammonium concentrations co-occurred with high oxygen–a hot moment. Nitrification rates are typically much lower, and on average, pelagic nitrification is expected to have only small or moderate effects on lake oxygen concentrations in winter. Nitrification rates, along with concentrations of NH_4_^+^ and NO_3_^-^ were related to N_2_O (Fig 5), a greenhouse gas which was consistently supersaturated across the systems during winter (Table 1). Supersaturation of N_2_O under ice suggests winter N_2_O accumulation should be considered, in addition to other greenhouse gases, to assess impacts on global greenhouse gas budgets.

## Supporting information

S1 Table Nitrification rates (following calculations outlined in [1]) for both 24 and 60 hours and associated other variables for this study (under ice cover) and for Lake St. George (near surface at 2 m depth, under ice cover; [3]); for Lake Superior (near surface at 2 m depth, in winter but without ice-cover; [4]) and [5]); and for surface estimates of nitrate accumulation in Wisconsin lakes part of the North Temperate Lakes Long-Term Ecological Research (NTL- LTER) study (ice-covered, 30 years of accumulated data; [6]). Values below LOQ for nitrification rates are reported, including negative values (following [4]) and sample-specific LOQ (calculated as per [7,8]) are reported. As noted in the main text, nitrification rates did not differ significantly based on incubation time. Despite this we caution that there may have been some recycling, hence 60 h incubations may underestimate nitrification rates.
(DOCX)
