# Supplementary material for: Winter nitrification in ice-covered lakes
Source: PLoS One. 2019 Nov 7;14(11):e0224864. doi: 10.1371/journal.pone.0224864 (PMC6837456; doi:10.1371/journal.pone.0224864)
Supplement: S1 Table — Values below LOQ for nitrification rates are reported, including negative values (following [4]) and sample-specific LOQ (calculated as per [7,8]) are reported. As noted in the main text, nitrification rates did not differ significantly based on incubation time. Despite this we caution that there may have been some recycling, hence 60 h incubations may underestimate nitrification rates. (DOCX) [file pone.0224864.s001.docx]

## Supplemental Information

S1 Table presents nitrification rates and other site data not included in Tables 1 and 2 of the main text. Nitrification rates were measured (modified from [1,2]) for both two different lengths of incubation (24 and 60h) in the winter of 2016 in order to account for the possibility of isotope recycling. While no statistically significant differences were found (see main text), we describe the 60h rates as minimum nitrification rates due to the tendency for these rates to be lower than rates derived from 24h incubations. Data presented the main text uses these 60h rates because the shorter duration incubation was not employed in the first study year.

S1 Table Nitrification rates (following calculations outlined in [1]) for both 24 and 60 hours and associated other variables for this study (under ice cover) and for Lake St. George (near surface at 2 m depth, under ice cover; [3]); for Lake Superior (near surface at 2 m depth, in winter but without ice-cover; [4]) and [5]); and for surface estimates of nitrate accumulation in Wisconsin lakes part of the North Temperate Lakes Long-Term Ecological Research (NTL- LTER) study (ice-covered, 30 years of accumulated data; [6]). Values below LOQ for nitrification rates are reported, including negative values (following [4]) and sample-specific LOQ (calculated as per [7,8]) are reported. As noted in the main text, nitrification rates did not differ significantly based on incubation time. Despite this we caution that there may have been some recycling, hence 60 h incubations may underestimate nitrification rates.

| Location | Study | Date | Incubation Duration | Mean depth | Surface Area | Nitrification Rate | Nitrification Rate LOQ | Temperature | Chlorophyll | Sulfate |
| --- | --- | --- | --- | --- | --- | --- | --- | --- | --- | --- |
|  |  |  | (h) | (m) | (km^2^) | (μg N L^-1^d^-1^) | (μg N L^-1^d^-1^) | (°C) | (ug L^-1^) | (mg L^-1^) |
| Blackstrap Reservoir | This Study | 05-Mar-15 | 60 | 5 | 12 | -1.7* | 4.9 x 10^-3^ | 2.36 | 4.9 | NA |
| Buffalo Pound Lake | This Study | 10-Mar-15 | 60 | 3 | 29.1 | -4.0* | 4.0 x 10^-3^ | 3.35 | 15 | 341 |
| Katepwa Lake | This Study | 10-Mar-15 | 60 | 14.3 | 16.2 | 32 | 2.7 x 10^-2^ | 0.42 | 0.31 | 516 |
| Pasqua Lake | This Study | 10-Mar-15 | 60 | 6 | 20.2 | 870.7 | 3.2 x 10^-3^ | 0.16 | 14 | 804 |
| Lenore Lake | This Study | 25-Mar-15 | 60 | 10 | 0.5 | -1.7* | 6.8 x 10^-3^ | 0.13 | 0.92 | 2048 |
| St. Brieux Lake | This Study | 25-Mar-15 | 60 | 6 | 1.9 | 110 | 1.8 x 10^-3^ | 0.59 | 0.25 | 738 |
| St. Denis Pond 1 | This Study | 16-Apr-15 | 60 | 1.3 | 0.1 | -0.042* | 3.2 x 10^-3^ | 5.35 | 23.1 | 653 |
| St. Denis Pond 5340 | This Study | 16-Apr-15 | 60 | 4.1 | 0.1 | -0.044* | 3.1 x 10^-3^ | 3.26 | 43 | 1457 |
| St. Denis Pond 90 | This Study | 16-Apr-15 | 60 | 3.7 | 0.21 | -0.10* | 3.6 x 10^-3^ | 7.02 | 12 | 738 |
| Echo Lake | This Study | 23-Feb-16 | 24 | 9.8 | 12.5 | 2.2 | 1.4 x 10^-1^ | 1.79 | 0.67 | 265 |
| Echo Lake | This Study | 23-Feb-16 | 60 | 9.8 | 12.5 | 1.2 | 7.0 x 10^-2^ | 1.79 | 0.67 | 265 |
| Mission Lake | This Study | 23-Feb-16 | 24 | 7.7 | 8.2 | 2.7 | 1.5 x 10^-1^ | 1.67 | 1.6 | 261 |
| Mission Lake | This Study | 23-Feb-16 | 60 | 7.7 | 8.2 | 2.1 | 1.1 x 10^-1^ | 1.67 | 1.6 | 261 |
| ELA Lake 227 | This Study | 14-Mar-16 | 24 | NA | NA | 0.41 | 9.5 x 10^-5^ | 0.78 | 11 | NA |
| ELA Lake 227 | This Study | 14-Mar-16 | 60 | NA | NA | 0.14 | 4.6 x 10^-5^ | 0.78 | 11 | NA |
| ELA Lake 239 | This Study | 14-Mar-16 | 24 | NA | NA | 0.18 | 1.8 x 10^-4^ | 0.42 | 0.57 | NA |
| ELA Lake 239 | This Study | 14-Mar-16 | 60 | NA | NA | 0.022 | 1.2 x 10^-4^ | 0.42 | 0.57 | NA |
| Lake St. George, Ontario | Knowles and Lean 1987 | 27-Feb-80 | NA | 17 | 0.0588 | 22.8 | NA | NA | NA | NA |
| Lake St. George, Ontario | Knowles and Lean 1987 | 06-Mar-80 | NA | 17 | 0.0588 | 12.2 | NA | NA | NA | NA |
| Lake St. George, Ontario | Knowles and Lean 1987 | 24-Feb-82 | NA | 17 | 0.0588 | 4.5 | NA | NA | NA | NA |
| Lake St. George, Ontario | Knowles and Lean 1987 | 03-Mar-82 | NA | 17 | 0.0588 | 1.3 | NA | NA | NA | NA |
| Lake St. George, Ontario | Knowles and Lean 1987 | 09-Feb-83 | NA | 17 | 0.0588 | 10.5 | NA | NA | NA | NA |
| Lake Croche | Masse et al. 2019 | 01-Feb-12 | NA | <12 max depth | 0.179 | 4.7 | NA | NA | NA | NA |
| Lake Croche | Masse et al. 2019 | 01-Mar-12 | NA | <12 max depth | 0.179 | 2.6 | NA | NA | NA | NA |
| Lake Croche | Masse et al. 2019 | 01-May-12 | NA | <12 max depth | 0.179 | 0.81 | NA | NA | NA | NA |
| Lake Croche | Masse et al. 2019 | 27-May-12 | NA | <12 max depth | 0.179 | 0.49 | NA | NA | NA | NA |
| Allequash Lake, Wisconsin NTL-LTER | Powers et al. 2017 | 30 years of data | NA | 2.9 | 1.12 | 0.58^ƒ^ | NA | NA | NA | NA |
| Big Musky Lake, Wisconsin NTL-LTER | Powers et al. 2017 | 30 years of data | NA | 7.5 | 3.96 | 0.55^ƒ^ | NA | NA | NA | NA |
| Crystal Lake, Wisconsin NTL-LTER | Powers et al. 2017 | 30 years of data | NA | 10.4 | 0.367 | 0.39^ƒ^ | NA | NA | NA | NA |
| Sparkling Lake, Wisconsin NTL-LTER | Powers et al. 2017 | 30 years of data | NA | 10.9 | 0.64 | 0.45^ƒ^ | NA | NA | NA | NA |
| Trout Lake, Wisconsin NTL-LTER | Powers et al. 2017 | 30 years of data | NA | 14.6 | 10.9 | 0.87^ƒ^ | NA | NA | NA | NA |
| Western Basin of Lake Superior | Small et al. 2013 | 11-Nov-09 | NA | 150 | NA | 0.34 | NA | NA | NA | NA |
| Western Basin of Lake Superior | Small et al. 2013 | 27-Jun-10 | NA | 150 | NA | 0.11 | NA | NA | NA | NA |
| Western Basin of Lake Superior | Small et al. 2013 | 19-Aug-10 | NA | 150 | NA | 0.33 | NA | NA | NA | NA |
| Western Basin of Lake Superior | Small et al. 2013 | 20-Mar-11 | NA | 150 | NA | 0.03 | NA | NA | NA | NA |
| *Indicates less than associated Limits of Quantitation for that sample. NA indicates data were not reported. ^ƒ^Indicates data from Powers et al. 2017 are estimated from winter nitrate accumulation and likely underestimated. | | | | | | | | | | |

## References for Supplemental Information

1. Sigman DMM, Altabet M A., Michener R, McCorkle DCC, Fry B, Holmes RMM. Natural abundance-level measurement of the nitrogen isotopic composition of oceanic nitrate: an adaptation of the ammonia diffusion method. Mar Chem. 1997;57: 227–242. doi:10.1016/S0304-4203(97)00009-1

2. Ward BB. Nitrogen transformations in the Southern California Bight. Deep Sea Res Part A Oceanogr Res Pap. 1987;34: 785–805. doi:10.1016/0198-0149(87)90037-9

3. Knowles R, Lean DRS. Nitrification: a significant cause of oxygen depletion under winter ice. Can J Fish Aquat Sci. 1987;44: 743–749.

4. Small GE, Bullerjahn GS, Sterner RW, Beall BFN, Brovold S, Finlay JC, et al. Rates and controls of nitrification in a large oligotrophic lake. Limnol Oceanogr. 2013;58: 276–286. doi:https://doi.org/10.4319/lo.2013.58.1.0276

5. Massé S, Botrel M, Walsh DA, Maranger R. Annual nitrification dynamics in a seasonally ice-covered lake. PLoS One. 2019;14: 1–21. doi:10.1371/journal.pone.0213748

6. Powers SM, Labou SG, Baulch HM, Hunt RJ, Lottig NR, Hampton SE, et al. Ice duration drives winter nitrate accumulation in north temperate lakes. Limnol Oceanogr Lett. 2017; 177–186. doi:10.1002/lol2.10048

7. Montoya JP, Voss M, Kahler P, Capone DG. A simple, high-precision, high-sensitivity tracer assay for N2 fixation. Appl Environ Microbiol. 1996;62: 986–993.

8. Peng X, Fuchsman CA, Jayakumar A, Warner MJ, Devol AH, Ward BB. Revisiting nitrification in the Eastern Tropical South Pacific: A focus on controls. J Geophys Res Ocean. 2016;121: 1667–1684. doi:10.1002/2015JC011455.Received
